# Identification of novel high-impact recessively inherited type 2 diabetes risk variants in the Greenlandic population

**DOI:** 10.1007/s00125-018-4659-2

**Published:** 2018-06-20

**Authors:** Niels Grarup, Ida Moltke, Mette K. Andersen, Peter Bjerregaard, Christina V. L. Larsen, Inger K. Dahl-Petersen, Emil Jørsboe, Hemant K. Tiwari, Scarlett E. Hopkins, Howard W. Wiener, Bert B. Boyer, Allan Linneberg, Oluf Pedersen, Marit E. Jørgensen, Anders Albrechtsen, Torben Hansen

**Affiliations:** 1 Novo Nordisk Foundation Center for Basic Metabolic Research, Faculty of Health and Medical Sciences, University of Copenhagen, Blegdamsvej 3B, 2200 Copenhagen, Denmark; 2 The Bioinformatics Centre, Department of Biology, University of Copenhagen, Ole Maaløes Vej 5, 2200 Copenhagen, Denmark; 3 National Institute of Public Health, University of Southern Denmark, Copenhagen, Denmark; 4 Greenland Centre for Health Research, University of Greenland, Nuuk, Greenland; 5 Department of Biostatistics, School of Public Health, University of Alabama at Birmingham, Birmingham, AL USA; 6 Center for Alaska Native Health Research, University of Alaska Fairbanks, Fairbanks, AK USA; 7 Department of Epidemiology, School of Public Health, University of Alabama at Birmingham, Birmingham, AL USA; 8 Center for Clinical Research and Prevention, Bispebjerg and Frederiksberg Hospital, The Capital Region, Copenhagen, Denmark; 9 Department of Clinical Medicine, Faculty of Health and Medical Sciences, University of Copenhagen, Copenhagen, Denmark; 10 Steno Diabetes Center Copenhagen, Gentofte, Denmark; 11 Faculty of Health Sciences, University of Southern Denmark, Odense, Denmark

**Keywords:** Genetic association, Genome-wide association study, Greenlanders, Inuit, *ITGA1*, *LARGE1*, Recessive genetic model, Type 2 diabetes

## Abstract

**Aims/hypothesis:**

In a recent study using a standard additive genetic model, we identified a *TBC1D4* loss-of-function variant with a large recessive impact on risk of type 2 diabetes in Greenlanders. The aim of the current study was to identify additional genetic variation underlying type 2 diabetes using a recessive genetic model, thereby increasing the power to detect variants with recessive effects.

**Methods:**

We investigated three cohorts of Greenlanders (B99, *n* = 1401; IHIT, *n* = 3115; and BBH, *n* = 547), which were genotyped using Illumina MetaboChip. Of the 4674 genotyped individuals passing quality control, 4648 had phenotype data available, and type 2 diabetes association analyses were performed for 317 individuals with type 2 diabetes and 2631 participants with normal glucose tolerance. Statistical association analyses were performed using a linear mixed model.

**Results:**

Using a recessive genetic model, we identified two novel loci associated with type 2 diabetes in Greenlanders, namely rs870992 in *ITGA1* on chromosome 5 (OR 2.79, *p* = 1.8 × 10^−8^), and rs16993330 upstream of *LARGE1* on chromosome 22 (OR 3.52, *p* = 1.3 × 10^−7^). The *LARGE1* variant did not reach the conventional threshold for genome-wide significance (*p* < 5 × 10^−8^) but did withstand a study-wide Bonferroni-corrected significance threshold. Both variants were common in Greenlanders, with minor allele frequencies of 23% and 16%, respectively, and were estimated to have large recessive effects on risk of type 2 diabetes in Greenlanders, compared with additively inherited variants previously observed in European populations.

**Conclusions/interpretation:**

We demonstrate the value of using a recessive genetic model in a historically small and isolated population to identify genetic risk variants. Our findings give new insights into the genetic architecture of type 2 diabetes, and further support the existence of high-effect genetic risk factors of potential clinical relevance, particularly in isolated populations.

**Data availability:**

The Greenlandic MetaboChip-genotype data are available at European Genome-Phenome Archive (EGA; https://ega-archive.org/) under the accession EGAS00001002641.

**Electronic supplementary material:**

The online version of this article (10.1007/s00125-018-4659-2) contains peer-reviewed but unedited supplementary material, which is available to authorised users.

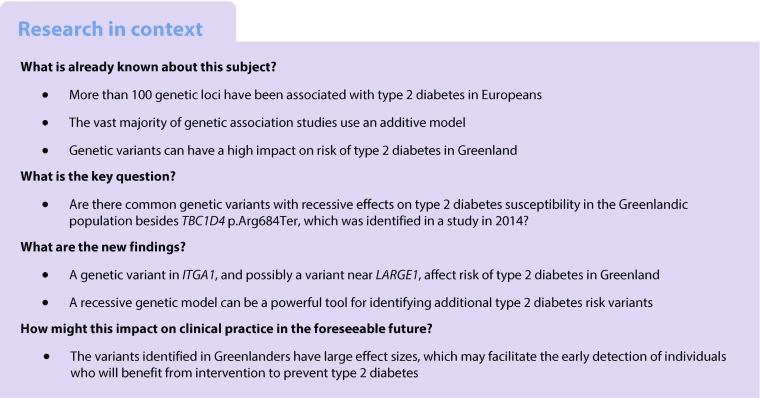



## Introduction

Numerous genome-wide association studies (GWAS) have been performed to identify genetic factors predisposing to type 2 diabetes. These studies, mainly performed in European and Asian populations, have identified around 120 genetic variants associated with risk of type 2 diabetes [1–8].

Until now, GWAS of type 2 diabetes and glycaemic traits have almost exclusively been performed using an additive genetic model [1–5]. This model, however, has limited statistical power to detect associations with variants displaying recessive effects [9, 10], unless the effect is very large. Recently, a GWAS of type 2 diabetes in up to 4040 individuals with type 2 diabetes and 116,246 participants without diabetes from the UK Biobank applied a dominance deviation model to identify non-additive association signals [11]. Although no novel signals were identified, the paper reported a recessive effect on risk of type 2 diabetes at the previously identified *CDKAL1* locus [11], which is in concordance with the findings from the original discovery [12]. Importantly, given the effective sample size of around 15,300, this study lacked statistical power to exclude the possibility that additional alleles with recessive effects predispose to type 2 diabetes in European populations.

We previously demonstrated the existence of a high-impact type 2 diabetes risk allele with predominately recessive effect in the Greenlandic population, despite using an additive genetic model in the discovery analysis [13]. Notably, however, this *TBC1D4* loss-of-function variant was discovered via analyses of a type 2 diabetes-related trait, 2 h plasma glucose, on which it had an extremely large effect, and not via analyses of type 2 diabetes. Indeed, the study in which that variant was discovered had very limited statistical power to detect variants with recessive effects on type 2 diabetes unless they were very frequent and had very large effects. Here, we aimed to identify additional variants with recessive effects on risk of type 2 diabetes in the Greenlandic population by applying a recessive genetic model to an increased number of Greenlandic samples with type 2 diabetes information.

## Methods

### Study populations

The Greenlandic study sample comprised individuals from two cohorts selected as part of a general population health survey of the Greenlandic population during the periods 1999–2001 (B99, *n* = 1401) and 2005–2010 (Inuit Health in Transition (IHIT), *n* = 3115) and from a cohort of Greenlanders living in Denmark on whom information was collected during 1998–1999 (BBH, *n* = 547) [14, 15]. There was an overlap of individuals (*n* = 295) between the IHIT and B99 cohorts, and these were assigned to B99. Of the 4674 genotyped individuals who passed quality control, 4648 had phenotype data available. The characteristics of the three cohorts are shown in electronic supplementary material (ESM) Table 1. All participants gave informed consent, and the study was approved by the Commission for Scientific Research in Greenland (project 2011-13, ref. no. 2011-056978; and project 2013-13, ref. no. 2013-090702), and conducted in accordance with the ethical standards of the Declaration of Helsinki, second revision.

Yup’ik samples for quantitative trait replication came from the Center for Alaska Native Health Research (CANHR), which performs studies related to genetic, behavioural, and nutritional risk factors for obesity and cardiometabolic diseases among Yup’ik people in a community-based setting [16]. Recruitment of Yup’ik families was initiated in 2003 and continues in 11 Southwest Alaska communities, where all residents are invited to participate, resulting in a convenience sample. The present study sample comprised 1059 non-pregnant Yup’ik individuals aged 14 years or above at the time of enrolment. All participants signed informed consent documents, and the study protocols were approved by the Institutional Review Boards of the University of Alaska and the National and Alaska Area Indian Health Service Institutional Review Boards, as well as the Yukon Kuskokwim Health Corporation Human Studies Committee.

Additional replication analyses were performed on up to 23,776 Danish samples from the Inter99 study (*n* = 4947) (CT00289237, ClinicalTrials.gov) [17], from Health2006–Health2010 studies (*n* = 4776) [18], from the Danish study of Functional Disorders (DanFunD; *n* = 6004) [19], from the Vejle Diabetes Biobank (*n* = 6266) [20], and from the Steno Diabetes Center Copenhagen (*n* = 1783). Characteristics of the cohorts have previously been published [6]. The studies were approved by the appropriate Regional Ethical Committees and were performed in accordance with the scientific principles of the Helsinki Declaration, second revision.

### Measurements and assays

The Greenlandic participants underwent an OGTT in which blood samples were drawn after an overnight fast and after 2 h during a 75 g OGTT. Plasma glucose levels were analysed using a Hitachi 912 system (Roche Diagnostics, Indianapolis, IN, USA), serum insulin with an immunoassay excluding des-31,32 split products and intact proinsulin (AutoDELFIA; PerkinElmer, Waltham, MA, USA), and HbA_1c_ by ion-exchange HPLC (G7 [Tosoh Bioscience, Tokyo, Japan] for IHIT samples; VARIANT [Bio-Rad, Hercules, CA, USA] for B99 samples). The thickness of visceral and subcutaneous adipose tissue was measured according to a validated protocol using a portable ultrasound scanner (Pie Medical, Maastricht, the Netherlands) with a 3.5 MHz transducer [21]. Serum cholesterol, HDL-cholesterol and triacylglycerol concentrations were measured using enzymatic calorimetric techniques (Hitachi 917, Roche Molecular Biochemicals, Indianapolis, IN, USA). LDL-cholesterol concentration was calculated according to Friedewald’s formula. Insulin resistance was estimated by either: (1) HOMA-IR [22], calculated as [fasting glucose level (mmol/l) × fasting insulin (pmol/l)/6.945]/22.5; or (2) the insulin sensitivity index, ISI(0,120) [23], calculated as [(75,000 + [fasting glucose (mmol/l) × (18 − 2 h glucose [mmol/l]) × 18] × 0.19 × weight [kg])/120] / ([fasting glucose (mmol/l) + 2 h glucose (mmol/l)]/2) / log([fasting insulin (pmol/l)/6.945] + [2 h insulin (pmol/l)/6.945]/2), where log is the natural logarithm.

In the CANHR Yup’ik study population, blood samples were collected after an overnight fast. Insulin was measured with a radioimmunoassay applying an ^125^I-iodinated insulin tracer, anti-human insulin-specific antibody and human insulin standards from Linco Research (Winchester, VA, USA). The Poly-Chem System Chemistry Analyzer (Polymedco, Courtlandt Manor, NY, USA) was applied to measure HDL-cholesterol, total cholesterol and triacylglycerols. Fasting blood glucose was measured on a Cholestech LDX analyser (Cholestech, Hayward, CA, USA), and glycosylated haemoglobin was measured on a Bayer HbA_1c_ DCA 2000+ analyser (Bayer, Leverkusen, Germany).

In the Danish samples, plasma glucose was measured by the glucose oxidase method (Granutest; Merck, Darmstadt, Germany).

In the Greenlandic and Danish cohorts, type 2 diabetes was defined based on self-reported type 2 diabetes, fasting plasma glucose level >7 mmol/l, or 2 h plasma glucose during an OGTT >11.1 mmol/l. Control individuals were normal glucose tolerant with a fasting plasma glucose <6.1 mmol/l and 2 h plasma glucose during an OGTT <7.8 mmol/l.

### Genotyping

The Greenlandic samples were genotyped using Illumina MetaboChip (Illumina, San Diego, CA, USA), which contains 196,725 SNPs potentially related to metabolic, cardiovascular or anthropometric traits [24]. Details about the genotyping procedure and quality control have previously been described [25]. In total, 4674 individuals (IHIT, 2791; B99, 1336; BBH, 547) and 115,182 SNPs passed the quality control.

Detailed descriptions of the genotyping procedures and data cleaning of CANHR Yup’ik samples have also previously been published [26]. Briefly, we used the Illumina Linkage IV panel to genotype 6090 SNPs spanning the entire genome, with an average genetic distance of 0.58 cM. A total of 5632 autosomal SNPs from this Linkage IV panel passed the quality control. These SNPs were used to obtain ancestry information for the statistical analysis. Additionally, genotyping of SNPs for replication was performed by the KASPar Genotyping assay (LGC Genomics, Hoddesdon, UK).

The Danish samples were genotyped by the Illumina Infinium OmniExpress-24 v1.1 array. Genotypes were called by the Illumina GenCall algorithm, and variants with a call rate <98% and Hardy–Weinberg equilibrium *p* < 1 × 10^−5^ were removed. Samples were excluded if they were ethnic outliers, had a mismatch between genetic and phenotypic sex or had a call rate <95%. Genotype data were imputed using the Haplotype Reference Consortium (HRC) reference panel v1.1 [27] at the Michigan imputation server using MiniMac3 v2.0.1 (https://genome.sph.umich.edu/wiki/Minimac3) after phasing with Eagle2 v2.4 (https://data.broadinstitute.org/alkesgroup/Eagle/) [28]. Post-imputation filtering of SNPs excluded variants with a minor allele frequency (MAF) <0.01 and info score <0.70.

### Statistical power simulations

To assess the statistical power of an additive and a recessive model-based test to detect association to a variant with recessive effect, we performed a range of simulations of data from a locus with a type 2 diabetes effect allele. For each of 540 combinations of effect allele frequencies (EAFs; EAF values 0.05, 0.1, 0.15, 0.2, 0.25 and 0.3) and effect sizes (ORs ranging from 1.1 to 10 with a step size of 0.1), we simulated first genotypes for *n* individuals and then their diabetes status based on these genotypes. For the simulation of type 2 diabetes status, we assumed a baseline risk of type 2 diabetes of 0.1, which we used to randomly simulate diabetes status for all individuals who were not homozygous carriers of the effect allele. For homozygous carriers of the effect allele, we simulated the diabetes status while taking both the baseline risk and the effect size into account. For each combination of EAF and OR, we performed 20,000 simulations, and finally, to estimate the power for the additive and the recessive model-based tests, we tested for association using logistic regression assuming each of these models, and estimated power as the proportion of tests that gave a *p* value <4.3 × 10^−7^ (the Bonferroni-corrected significance threshold of this study; see below for details). We performed the power simulations for 2948 individuals to reflect the number of individuals with type 2 diabetes information available in this study*.*

### Statistical association analysis

For analyses of association in the Greenlanders, we used a linear mixed model, implemented in GEMMA software v0.96 (http://www.xzlab.org/) [29]. This model controls for admixture and relatedness between individuals as random effects via a genetic similarity matrix, which we estimated using GEMMA. This control is needed because the Greenlandic population is admixed with ancestry from both Inuit and Europeans [30]. With this model, we achieved an acceptable genomic inflation factor (λ = 1.009; ESM Fig. 1). In the discovery analyses, we used a recessive genetic model and applied a study-wide significance threshold of *p* = 4.3 × 10^−7^ corresponding to a Bonferroni correction for analysing 115,182 SNPs. In all the association analyses, we analysed data combined from the three cohorts and included sex, age and cohort as covariates. Prior to performing the association tests, quantitative traits were quantile-transformed to a standard normal distribution within each sex and each cohort. Therefore, for quantitative traits, effect sizes are reported as β in SD units (95% CI) in the text, and additionally as trait units in tables, obtained from association analyses on raw trait values. Effect sizes for association with type 2 diabetes are reported as ORs in the text, and were obtained from logistic regression analyses adjusted for age, sex and ten principal components. We estimated the effect sizes for the Inuit and European ancestry components using logistic regression also adjusted for age, sex and ten principal components with asaMap (https://github.com/ANGSD/asaMap, accessed 1 August 2017) [31] based on the estimated admixture proportions.

The Yup’ik cohort was also analysed using GEMMA software [29]. Here, however, the genetic similarity matrix required for the association analysis was not estimated using GEMMA, as we did in our analyses of the Greenlanders, but was instead calculated using the genotype data from the linkage panel merged with the additional genotypes of the SNPs genotyped for this study.

In the Danish data, association analyses of type 2 diabetes were carried out by logistic regression adjusting for age, sex and the first ten principal components. These analyses were performed on imputed genotype data, taking genotype uncertainty into account, applying the expected count test in SNPTEST v2.5.2 (https://mathgen.stats.ox.ac.uk/genetics_software/snptest/snptest.html).

### Allele frequency estimation

For the SNPs of interest, we estimated allele frequencies for each of the Inuit and European ancestry components of the Greenlandic population using a two-step approach. In step 1, we estimated ancestry proportions for the Greenlandic study individuals as well as 50 Danish individuals. To do this, we applied ADMIXTURE v1.3.0 (https://www.genetics.ucla.edu/software/admixture/) [32] to all the SNP data from these individuals, assuming two ancestral populations—Inuit and Europeans. In step 2, we estimated ancestral allele frequencies with CIs for each SNP separately using bootstrap with replacement. We used 1000 bootstrap samples of individuals and, based on each, we performed maximum likelihood estimation of the allele frequencies, using the likelihood from ADMIXTURE with the ancestry proportions fixed to the estimates obtained in step 1. The CIs were based on the quantiles of these bootstrap estimates.

In CANHR Yup’ik samples, allele frequencies for the SNPs of interest were estimated using the Mendel program v16 (https://www.genetics.ucla.edu/software/mendel) [33, 34].

### Querying results from previous large GWAS of type 2 diabetes and metabolic traits

Additive genetic model GWAS results were queried online: type 2 diabetes results from the DIAGRAM Consortium (DIAbetes Genetics Replication And Meta-analysis; http://diagram-consortium.org) [8], glycaemic trait results from MAGIC (the Meta-Analyses of Glucose and Insulin-related traits; www.magicinvestigators.org) [35], anthropometric trait results from the GIANT Consortium (Genetic Investigation of Anthropometric Traits; http://portals.broadinstitute.org/collaboration/giant/index.php/GIANT_consortium) [36–38], and lipid results from GLGC (Global Lipids Genetics Consortium; http://lipidgenetics.org) [39].

### Assessment of functional effects and expression quantitative trait loci

To investigate whether the associated variants were causal, RegulomeDB (http://www.regulomedb.org/, accessed 10 January 2018) and HaploReg v4.1 (http://archive.broadinstitute.org/mammals/haploreg/haploreg.php) were applied to assess possible co-localisation of the genetic variants with regulatory elements, such as transcription factor binding sites, promoter regions and regions of DNAase hypersensitivity. We also used data from the GTEx Consortium available online to investigate associations between genetic variants and RNA expression (www.gtexportal.org; accessed 27 January 2018). All 48 tissues with more than 70 samples were queried. Sample sizes ranged from 80 to 388. Results were only available for an additive genetic model.

## Results

**Fig. 1:**
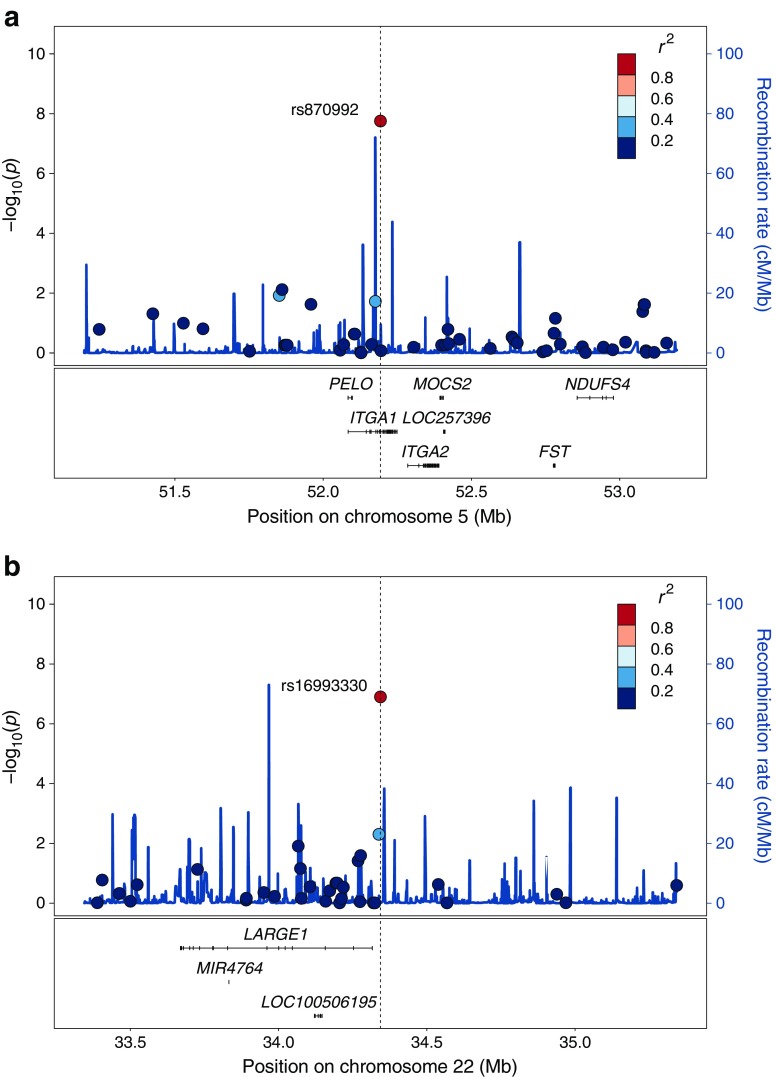
Association results with type 2 diabetes for those SNPs on the MetaboChip that are located in a 2 Mb region around the lead SNPs: (**a**) rs870992 in *ITGA1*, and (**b**) rs16993330 near *LARGE1*. Each SNP in the region is represented by a circle whose colour indicates the extent of correlation (*r*^2^) between the SNP and the lead SNP, which is shown in red. The position of the circle along the *x*-axis shows the genomic position of the SNP. The position of the circle along the left *y*-axis shows the −log_10_ (*p*) value of the SNP when testing for association with type 2 diabetes as determined using a recessive model. The solid blue line illustrates the recombination rate from the Chinese HapMap Phase III panel (www.sanger.ac.uk/resources/downloads/human/hapmap3.html). The protein-coding genes in the genetic region are shown below the plot

We analysed association with type 2 diabetes for 317 individuals with type 2 diabetes and 2631 participants with normal glucose tolerance. Using a recessive genetic model on these Greenlandic data, we identified three loci associated with type 2 diabetes below the study-wide Bonferroni-corrected significance threshold (*p* < 4.3 × 10^−7^) (ESM Fig. 1). Besides the previously described *TBC1D4* locus [13], these comprised two novel loci, namely the common intron variant rs870992 in *ITGA1* on chromosome 5 (MAF 23%, OR 2.79, *p* = 1.8 × 10^−8^) and the common intergenic variant rs16993330 approximately 25 kb upstream of *LARGE1* on chromosome 22 (MAF 16%, OR 3.52, *p* = 1.3 × 10^−7^) (Fig. 1, Table 1). However, the *LARGE1* variant did not reach the conventional threshold for genome-wide significance (*p* < 5 × 10^−8^). Similar associations with type 2 diabetes were observed when adjusting for BMI (data not shown).

**Table 1:** Variants on MetaboChip associated with type 2 diabetes in Greenlanders under a recessive genetic model

			EAF (95% CI)			Type 2 diabetes							
						Recessive model				Additive model			
Locus	Chr	EA	All	Inuit	EU	β (95% CI)	*p* value	OR_All_	OR_Inuit_	β (95% CI)	*p* value	OR_All_	OR_Inuit_
*TBC1D4* rs7330796^a^	13	T	0.22 (0.21, 0.23)	0.27 (0.25, 0.29)	0.10 (0.08, 0.13)	0.18 (0.13, 0.23)	1.0 × 10^−14^	4.43	5.58	0.043 (0.03, 0.06)	1.5 × 10^−6^	1.67	1.80
*ITGA1* rs870992	5	G	0.23 (0.22, 0.24)	0.31 (0.30, 0.33)	0.03 (0.01, 0.06)	0.12 (0.079, 0.17)	1.8 × 10^−8^	2.79	3.12	0.029 (0.01, 0.05)	1.2 × 10^−3^	1.41	1.49
*LARGE1* rs16993330	22	A	0.16 (0.15, 0.17)	0.19 (0.18, 0.21)	0.07 (0.05, 0.09)	0.17 (0.11, 0.23)	1.3 × 10^−7^	3.52	3.54	0.028 (0.01, 0.05)	6.0 × 10^−3^	1.35	1.45

Analyses of type 2 diabetes comprised 317 individuals with type 2 diabetes and 2631 control participants with normal glucose tolerance. ORs were estimated using logistic regression and reported for the entire Greenlandic study sample (OR_All_) and the Inuit ancestry component (OR_Inuit_). EAFs are given for the entire Greenlandic study sample (All), as well as the Inuit ancestry component (Inuit), and the European ancestry component (EU) of the Greenlandic study population

^a^rs7330796 is the discovery variant for *TBC1D4* on the MetaboChip, not the causal variant [13]

Chr, chromosome; EA, effect allele

**Fig. 2:**
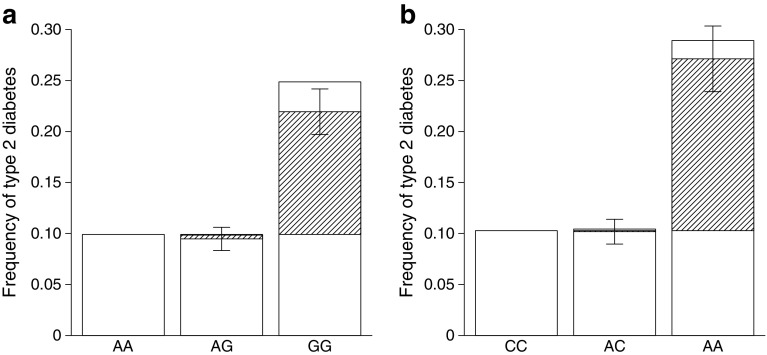
Frequency of type 2 diabetes among Greenlanders stratified by the genotypes of (**a**) *ITGA1* rs870992 and (**b**) *LARGE1* rs16993330. The superimposed hatched areas represent effect sizes and the error bars represent SE. The effect sizes and SEs were estimated using a linear mixed model with no assumptions of inheritance mode. Frequencies were calculated in the subset of individuals included in the type 2 diabetes case–control study

The Greenlandic population is admixed with ancestry from both Inuit and Europeans [30]. Interestingly, the estimated EAF were considerably higher in the Inuit ancestry component for *ITGA1* rs870992 (31%) and for *LARGE1* rs16993330 (19%), than in the European ancestry component among Greenlanders (3% and 7%, respectively) and in European populations (Table 1, ESM Table 2). The allele frequencies found in Europeans were so low that homozygous carriers were almost absent. The novel variants in *ITGA1* and *LARGE1* had a high impact on risk of type 2 diabetes in Greenlanders, evident by the estimated effect sizes (Table 1) and by the higher frequency of type 2 diabetes among homozygous carriers compared with non-carriers (24.9% and 28.9% vs 10%; Fig. 2). To gain further insights into the effect of the identified loci, we also performed type 2 diabetes association analyses of these assuming an additive model (Table 1). This led to low *p* values as well, although none of them was low enough to pass the Bonferroni-corrected significance threshold (*p* < 4.3 × 10^−7^). Based on genetic data and functional annotation analyses facilitated by in silico tools, we were unable to determine whether the identified variants were causal.

Next, to elucidate the diabetes-related mechanisms underlying the association of the novel variants, we analysed diabetes-related traits in individuals without diabetes from the Greenlandic cohorts with a recessive genetic model. Homozygous carriers of *ITGA1* rs870992 had nominally increased HbA_1c_ levels (β = 0.13 SD [95% CI 0.027, 0.23], *p* = 0.012) (Table 2). For *LARGE1* rs16993330, the underlying physiological mechanism seems to be linked to increased fat accumulation, indicated by higher levels of visceral adipose tissue (0.29 SD [95% CI 0.076, 0.50], *p* = 0.0078), larger hip circumference (0.22 SD [95% CI 0.048, 0.40], *p* = 0.013) and waist circumference (0.21 SD [95% CI 0.041, 0.38], *p* = 0.015) and higher BMI (0.18 SD [95% CI 0.0046, 0.35], *p* = 0.045) (Table 2). Furthermore, homozygous *LARGE1* rs16993330 carriers had increased insulin resistance, indicated by higher levels of fasting serum insulin (0.22 SD [95% CI 0.027, 0.41], *p* = 0.026) and HOMA-IR (0.24 SD [95% CI 0.048, 0.44], *p* = 0.014), and lower levels of the ISI (−0.21 SD [95% CI −0.016, −0.41], *p* = 0.034) (Table 2). We then performed the analyses of the same quantitative diabetes-related traits with an additive model and obtained similar results (ESM Table 3).

**Table 2:** Association of *ITGA1* rs870992 and *LARGE1* rs16993330 with quantitative metabolic traits in Greenlanders under a recessive genetic model

		*ITGA1* rs870992				*LARGE1* rs16993330			
Trait (measured unit)	*n*	β_SD_	95% CI_SD_	β	*p* value	β_SD_	95% CI_SD_	β	*p* value
Fasting plasma glucose (mmol/l)	3693	0.12	−0.0034, 0.25	0.16	0.059	0.15	−0.028, 0.32	0.19	0.10
2 h plasma glucose (mmol/l)	3437	0.070	−0.063, 0.20	0.36	0.31	0.12	−0.069, 0.31	0.32	0.21
Fasting serum insulin (pmol/l)	3691	0.016	−0.12, 0.16	1.9	0.82	0.22	0.027, 0.41	5.5	0.026
2 h serum insulin (pmol/l)	3437	0.0090	−0.13, 0.15	−5.0	0.91	0.16	−0.037, 0.36	6.6	0.11
HbA_1c_ (mmol/mol)	4626	0.13	0.027, 0.23	NA	0.012	−0.03	−0.17, 0.11	NA	0.67
HbA_1c_ (%)	4624	0.13	0.027, 0.23	0.063	0.012	−0.03	−0.17, 0.11	−0.011	0.67
HOMA-IR	3684	0.053	−0.086, 0.19	0.15	0.45	0.24	0.048, 0.44	0.33	0.014
ISI(0,120)	3404	−0.051	−0.19, 0.088	0.12	0.47	−0.21	−0.41, −0.016	−0.31	0.034
Weight (kg)	4631	−0.045	−0.17, 0.077	−0.59	0.47	0.20	0.031, 0.37	3.4	0.020
BMI (kg/m^2^)	4626	−0.078	−0.20, 0.047	−0.28	0.22	0.18	0.0046, 0.35	1.3	0.045
Waist circumference (cm)	4594	−0.054	−0.18, 0.068	−0.67	0.39	0.21	0.041, 0.38	3.3	0.015
Hip circumference (cm)	4592	−0.036	−0.16, 0.087	−0.37	0.57	0.22	0.048, 0.40	2.4	0.013
WHR	4591	−0.041	−0.15, 0.073	−0.0040	0.48	0.12	−0.041, 0.28	0.011	0.14
Visceral adipose tissue (cm)	2693	0.0020	−0.15, 0.15	0.031	0.98	0.29	0.076, 0.50	0.73	0.0078
Subcutaneous adipose tissue (cm)	2683	−0.090	−0.24, 0.065	−0.16	0.26	0.20	−0.018, 0.42	0.29	0.070
Fasting serum total cholesterol (mmol/l)	4517	−0.012	−0.13, 0.11	−0.0080	0.84	0.1	−0.058, 0.27	0.13	0.20
Fasting serum HDL-cholesterol (mmol/l)	4652	0.011	−0.11, 0.13	0.028	0.86	0.051	−0.11, 0.22	0.039	0.54
Fasting serum LDL-cholesterol (mmol/l)	3957	−0.014	−0.14, 0.11	−0.018	0.83	0.061	−0.11, 0.23	0.073	0.48
Fasting serum triacylglycerol (mmol/l)	4124	−0.034	−0.16, 0.095	0.0060	0.61	0.15	−0.022, 0.33	0.021	0.088

Analyses were performed using a recessive genetic model. β_SD_ is the effect size estimated from quantile-transformed values of the trait, and β is the effect size estimated from untransformed values. Values of *p* were obtained from the quantile-transformation based analyses

NA, not available

We subsequently attempted to validate our findings in another Arctic indigenous population by genotyping the *ITGA1* and *LARGE1* variants in 1059 Alaska Native Yup’ik. However, none of the potential associations with diabetes-related quantitative traits were replicated in this smaller sample (ESM Table 4), and analysis of type 2 diabetes was precluded due to the low number of participants with diabetes. A second line of replication was attempted in a Danish sample of 5220 individuals with type 2 diabetes and 18,556 control participants. However, we did not observe an association with type 2 diabetes using a recessive model (ESM Table 2). In addition, the results of a dominance deviance model GWAS of European UK Biobank samples [11] showed no significant rejection of the additive model for either *ITGA1* rs870992 or *LARGE1* rs16993330 (*p* = 0.54 and *p* = 0.36, respectively) based on 117,775 individuals. We also queried the two variants from the available summary data from a recent additive genetic model GWAS of type 2 diabetes [8] and found that the *ITGA1* rs870992 G allele was nominally associated with increased risk of type 2 diabetes, whereas no effect was observed for *LARGE1* rs16993330 (ESM Table 5). Additionally, queries of results from GWAS of other traits revealed associations of the *ITGA1* rs870992 G allele with increased total cholesterol and LDL-cholesterol levels, and nominal associations of the *LARGE1* rs16993330 A allele with increased total cholesterol and triacylglycerol levels (ESM Table 5). Owing to the possible inflation of the test statistics, we also included the quantile of *p* values for the significant tests in ESM Table 5.

Finally, we queried RNA expression data available online as part of the GTEx project (www.gtexportal.org/) under the hypothesis that the newly identified variants in *ITGA1* and *LARGE1* could change the expression of regional genes. Here we observed that the type 2 diabetes-associated *ITGA1* rs870992 G allele was associated with increased *ITGA1* RNA expression in nerve, pancreas and smooth muscle tissue in up to 361 samples, albeit with modest *p* values (*p* < 0.0001). No expression quantitative trait loci (eQTL) associations were found for *LARGE1* rs16993330.

## Discussion

Using a recessive genetic model, we investigated the association between markers on MetaboChip and type 2 diabetes in a Greenlandic study population. Besides the established *TBC1D4* locus [13], we identified a novel genome-wide significant variant for risk of type 2 diabetes in *ITGA1*, and a variant near *LARGE1* showing a suggestive association. Additional analyses of diabetes-related quantitative traits indicated that the *LARGE1* variant might increase the risk of type 2 diabetes through accumulation of visceral fat and increased insulin resistance, whereas these analyses provided no clues to the mechanistic link underlying the *ITGA1* association. This study demonstrates the value of using the correct genetic model for identification of disease-associated variants, which is also supported by simulations showing that, for example, for a recessive effect allele with a frequency of 0.25 and an OR of 3 (similar to the *ITGA1* variant), the power under an additive model is less than 20% whereas the power under a recessive model is around 80% (ESM Fig. 2). Moreover, under a wide range of EAF and ORs, the recessive model has markedly higher statistical power to detect variants with recessive effects associated with type 2 diabetes.

A possible link between the *ITGA1* locus and altered glucose regulation is supported by studies of type 2 diabetes-related traits. Recently, rs6450057 mapping to *PELO*, a gene embedded in intron 1 of *ITGA1*, was found to be associated with fasting serum insulin levels in a transethnic GWAS meta-analysis of 70,000 individuals [40]. Interestingly, this study found an opposite direction of effect in European and African-American samples, indicating that rs6450057 is not the causal variant at this locus. Moreover, another *ITGA1* intron variant, rs6867040, has been associated with fasting plasma glucose concentrations in 46,262 European individuals [41], and a recent prepublished GWAS of more than 800,000 samples showed an independent association between three variants at the *ITGA1* locus and type 2 diabetes using an additive genetic model [42]. These studies support our findings of a link between variation in *ITGA1* and type 2 diabetes. However, it is unclear whether it is the same signal, as the identified variants in the European study do not include rs870992, nor are the identified variants in high linkage disequilibrium with rs870992 in Europeans (*r*^2^ < 0.1).

Biologically, *ITGA1* is an attractive candidate gene, encoding the α1-integrin subunit, which heterodimerises with the β1 subunit to form cell surface receptors that bind collagen and laminin. Interestingly, α1β1-integrin is the primary collagen receptor used by cultured beta cells, and this interaction regulates beta cell adhesion, motility and insulin secretion [43, 44]. Moreover, specific interactions between α1β1-integrin and extracellular matrix have been shown to be critical for beta cell survival and function [45], and therefore are possibly also important for glucose homeostasis and risk of type 2 diabetes. Besides the possible effects on beta cell function, ITGA1 seems to be linked to liver function. Thus, *ITGA1* variants have been associated with plasma levels of the liver enzyme γ-glutamyl transferase [46], and modestly with ITGA1 protein expression levels in the liver [41]. In mice, knocking out *Itga1* leads to severe hepatic insulin resistance [47] and altered fatty acid metabolism when they are fed a high-fat diet [48]. In line with this, we observed a borderline significant association between *ITGA1* rs870992 and increased levels of fasting plasma glucose, which might indicate altered hepatic glucose regulation in the fasted state. Thus, studies of phenotypes reflecting liver function in Greenlanders would be of great interest to elucidate the biological mechanisms underlying the association between variation in *ITGA1* and type 2 diabetes. In eQTL analyses, no effect of the type 2 diabetes-associated rs870992 variant on mRNA expression in liver tissue was observed. Instead, the variant was associated with increased *ITGA1* expression in three other tissues; however, this was in the opposite direction to what was expected from the *Itga1* knockout mice. This difference might rely on the fact that rs870992 is unlikely to be the causal variant and therefore we cannot conclude that the causal variant acts through RNA regulation even if our top SNP is significantly associated with *ITGA1* expression.

No previous studies have indicated a link between *LARGE1* and type 2 diabetes in additive model GWAS [8], nor did we replicate our findings in recessive analysis in a Danish sample of 5220 individuals with diabetes and 18,556 control participants. This lack of association in European samples may rely on low statistical power for recessive effects, due to the low frequency of risk alleles (EAF<10%) in Europeans (ESM Fig. 2) or due to population-specific differences in linkage disequilibrium between the identified variant and the causal variant. It is also possible that the causal variant behind the observed type 2 diabetes association is Inuit-specific, similar to the risk variant in *TBC1D4* [13]. However, we were unable to pinpoint such a causal variant. Moreover, the lack of replication of the diabetes-related quantitative traits associations in a small sample of Alaska Native Yup’ik illustrates one of the main challenges with studies of isolated populations, namely the difficulty of finding appropriate replication cohorts in terms of both genetic composition and sample size. It is, however, also possible that the *LARGE1* association represents a false-positive discovery.

*LARGE1* encodes the LARGE xylosyl- and glucuronyltransferase 1 protein. This protein interacts with α-dystroglycan and functions as a glycosyltransferase stimulating glycosylation of α-dystroglycan, which in skeletal muscles connects the cytoplasm with the extracellular matrix as part of the dystrophin–glycoprotein complex [49, 50]. How this relates to the observed association with visceral fat accumulation and insulin resistance is unclear; therefore studies including a more comprehensive coverage of the genome and a larger sample size are warranted to verify the suggested associations, and possibly identify the causal variant in the locus.

In this study, we observed three loci, *TBC1D4, ITGA1* and *LARGE1*, harbouring variants with large recessive effects on the risk of type 2 diabetes in Greenlanders, the latter, however, having weaker statistical support. In addition to these loci, based on a loss-of-function screening of the same population, we recently identified a variant in *ADCY3* recessively associated with obesity and type 2 diabetes [51]. Variants in all four loci have effect sizes that are much greater than what has been reported for *TCF7L2*, which is the greatest genetic risk factor for type 2 diabetes among Europeans [1]. Even though the estimated effects are likely to be inflated by the ‘winner’s curse’, these variants have the potential to be of clinical relevance by facilitating the prediction of diabetes development in Greenlandic population. More research is needed to identify the causal variants, to elucidate the biological mechanisms underlying the association with type 2 diabetes, and to clarify whether these novel variants distinguish type 2 diabetes subtypes with specific aetiology, as demonstrated for *TBC1D4* [13]. This additional knowledge could have the potential to guide choice of treatment for subtypes of type 2 diabetes.

In conclusion, we demonstrate that common alleles with recessive effects play a role in the genetic architecture of type 2 diabetes in the small and historically isolated Greenlandic population. These findings reiterate the importance of considering non-additive genetic models when performing GWAS.

## Electronic supplementary material

ESM (PDF 510 kb)

## Data Availability

The Greenlandic MetaboChip-genotype data are available at European Genome-Phenome Archive (EGA; https://ega-archive.org/) under the accession EGAS00001002641.

## Abbreviations

CANHR: Center for Alaska Native Health Research
EAF: Effect allele frequency
eQTL: Expression quantitative trait loci
IHIT: Inuit Health in Transition
GWAS: Genome-wide association study/studies
ISI: Insulin sensitivity index
MAF: Minor allele frequency
