# Supplementary material for: Identification of novel high-impact recessively inherited type 2 diabetes risk variants in the Greenlandic population
Source: Diabetologia. 2018 Jun 20;61(9):2005–15. doi: 10.1007/s00125-018-4659-2 (PMC6096637; doi:10.1007/s00125-018-4659-2)
Supplement: Supplementary file 1 — (PDF 510 kb) [file 125_2018_4659_MOESM1_ESM.pdf]

Electronic Supplementary Material (ESM)

Identification of novel high-impact recessively inherited type 2 diabetes risk variants in Greenlandic Inuit

Niels Grarup[1]*, Ida Moltke[2]*, Mette K. Andersen[1]*, Peter Bjerregaard[3,4], Christina V.L. Larsen[3,4], Inger K. Dahl-Petersen[3], Emil Jørsboe[2], Hemant K. Tiwari[5], Scarlett E. Hopkins[6], Howard W. Wiener[7], Bert B. Boyer[6], Allan Linneberg[8,9], Oluf Pedersen[1], Marit E. Jørgensen[3,4,10], Anders Albrechtsen[2], Torben Hansen[1,11]

SUPPLEMENTARY TABLES

ESM Table 1. Description of Greenlandic cohorts

	IHIT			B99			BBH	
	**All**	**T2D-controls**	**T2D-cases**	**All**	**T2D-controls**	**T2D-cases**	**All**	**T2D-cases**
n (men/women)	2775 (1239/1536)	1885 (816/1069)	222 (103/119)	1332 (580/752)	746 (301/445)	82 (33/49)	541 (143/398)	13 (4/9)
Age (years)	44 (15)	40 (14)	58 (13)	44 (14)	43 (12)	57 (12)	43 (12)	53 (12)
Fasting plasma glucose (mmol/l)	5.7 (0.84)	5.4 (0.38)	7.3 (2)	5.8 (1.1)	5.4 (0.47)	8.3 (2.7)		
2h plasma glucose (mmol/l)	5.9 (2.4)	5.0 (1.2)	11 (4.1)	6.0 (2.9)	5.0 (1.3)	11 (6.4)		
Fasting serum insulin (pmol/l)	37 (25-56)	36 (24-52)	47 (32-82)	38 (26-57)	35 (25-51)	70 (43-120)		
2h serum insulin (pmol/l)	110 (48-210)	91 (40-170)	250 (130-430)	98 (43-200)	72 (36-150)	220 (80-360)		
HbA_{1C} (mmol/mol)	38 (5.0)	37 (3.5)	44 (10)	43 (6.9)	42 (4.9)	54 (16)	42 (6.0)	53 (11)
HbA_{1C} (%)	5.7 (0.46)	5.6 (0.32)	6.2 (0.92)	6.1 (0.63)	6 (0.44)	7.1 (1.5)	5.9 (0.55)	7.0 (1.0)
HOMA-IR	1.3 (0.87-2)	1.2 (0.82-1.8)	2.2 (1.3-3.6)	1.4 (0.91-2.1)	1.2 (0.82-1.8)	3.5 (2-6.2)		
ISI(0,120)	2.6 (1.9-3.9)	3.0 (2.3-4.3)	1.2 (0.95-1.6)	2.7 (1.9-4.2)	3.2 (2.4-4.7)	1.2 (0.96-1.9)		
BMI (kg/m^2)	26 (5.2)	26 (4.8)	29 (6.6)	26 (4.9)	26 (4.4)	31 (7.1)	25 (4.3)	28 (6.8)
Waist circumference (cm)	92 (14)	90 (12)	99 (16)	89 (13)	87 (12)	100 (16)	83 (12)	92 (16)
WHR	0.92 (0.079)	0.91 (0.077)	0.97 (0.084)	0.90 (0.084)	0.89 (0.078)	0.97 (0.094)	0.85 (0.08)	0.89 (0.091)
Visceral adipose tissue (cm)	7.0 (2.3)	6.7 (2)	8.3 (2.9)					
Subcutaneous adipose tissue (cm)	3.0 (1.5)	3.0 (1.5)	3.1 (1.5)					
Fasting serum total cholesterol (mmol/l)*	5.8 (1.2)	5.7 (1.2)	6.3 (1.3)	5.9 (1.2)	6 (1.1)	6.3 (1.2)	5.9 (1.2)	6.2 (1.1)
Fasting serum HDL-cholesterol (mmol/l)*	1.7 (0.55)	1.7 (0.5)	1.8 (0.82)	1.6 (0.46)	1.6 (0.44)	1.6 (0.6)	1.7 (0.51)	1.5 (0.58)
Fasting serum LDL-cholesterol (mmol/l)	3.6 (1.1)	3.5 (1.1)	3.7 (1.1)	3.8 (1.1)	3.9 (1.1)	4.0 (1.1)		
Fasting serum triacylglycerol (mmol/l)	1 (0.76-1.4)	0.98 (0.74-1.3)	1.2 (0.84-1.8)	0.98 (0.74-1.4)	0.9 (0.7-1.2)	1.4 (0.87-2.2)		
Type 2 diabetes (*n* (%))	222 (8)			82 (6.2)			13 (2.4)	
Non-diabetic control subjects (*n* (%))	1885 (68)			746 (56)			0 (0)	

Data are mean (standard deviation) or median (interquartile range) for non-normally distributed traits. * Non-fasting levels in the BBH cohort.

ESM Table 2. Association analyses of *ITGA1* rs870992 and *LARGE* rs16993330 in Danish samples applying a recessive genetic model

**Locus**	**Chr.**	**Alleles (EA/non-EA)**	**EAF**	***n* (wt/he/ho) for cases and controls**	**OR (95% CI)**	***p***
ITGA1 rs870992	5	G/A	0.077	Cases: 4444/737/38<br>Controls: 15798/2656/101	1.36 (0.93-1.95)	0.12
LARGE1 rs16993330	22	A/C	0.060	Cases: 4592/611/16<br>Controls: 16395/2078/82	0.69 (0.41-1.19)	0.32

EA, effect allele; EAF, effect allele frequency; he, heterozygous variant carriers; ho, homozygous variant carriers; wt, wild type variant carriers.

ESM Table 3. Association of *ITGA1* rs870992 and *LARGE1* rs16993330 with quantitative metabolic traits in Greenlanders under an additive genetic model

		ITGA1 rs870992				*LARGE1* rs16993330			
**Trait (measured unit)**	***n***	**β_{SD}**	**95% CI_{SD}**	**β**	***p***	**β_{SD}**	**95% CI_{SD}**	**β**	***p***
Fasting plasmaglucose (mmol/l)	3693	0.038	-0.015 to 0.091	0.047	0.16	0.038	-0.021 to 0.097	0.047	0.20
2h plasma glucose (mmol/l)	3437	0.034	-0.021 to 0.089	0.12	0.22	0.027	-0.034 to 0.088	0.091	0.39
Fasting serum insulin (pmol/l)	3691	0.014	-0.043 to 0.071	1.7	0.63	0.090	0.027 to 0.15	2.2	**0.0053**
2h serum insulin (pmol/l)	3437	0.028	-0.029 to 0.085	7.3	0.34	0.061	-0.0037 to 0.13	11	0.065
HbA_{1C} (mmol/mol)	4626	0.027	-0.016 to 0.07	NA	0.21	0.019	-0.028 to 0.066	NA	0.42
HbA_{1C} (%)	4624	0.027	-0.016 to 0.07	0.015	0.21	0.019	-0.028 to 0.066	0.016	0.42
HOMA-IR	3684	0.023	-0.034 to 0.08	0.062	0.42	0.094	0.031 to 0.16	0.13	**0.0036**
ISI(0,120)	3404	-0.035	-0.092 to 0.022	0.024	0.22	-0.069	-0.13 to -0.0063	-0.093	**0.033**
Weight (kg)	4631	-0.009	-0.06 to 0.042	-0.071	0.73	0.079	0.022 to 0.14	1.3	**0.006**
BMI (kg/m^2)	4626	-0.016	-0.069 to 0.037	-0.04	0.56	0.066	0.0092 to 0.12	0.42	**0.025**
Waist circumference (cm)	4594	-0.006	-0.057 to 0.045	-0.015	0.80	0.084	0.029 to 0.14	1.2	**0.003**
Hip circumference (cm)	4592	0.01	-0.041 to 0.061	0.12	0.70	0.055	-0.0018 to 0.11	0.56	0.058
WHR	4591	-0.015	-0.062 to 0.032	-0.002	0.54	0.083	0.03 to 0.14	0.008	**0.0018**
Visceral adipose tissue (cm)	2693	0.027	-0.036 to 0.09	0.075	0.41	0.064	-0.0085 to 0.14	0.18	0.081
Subcutaneous adipose tissue (cm)	2683	-0.04	-0.11 to 0.027	-0.054	0.23	0.049	-0.025 to 0.12	0.061	0.20
Fasting serum total cholesterol (mmol/l)	4517	0.03	-0.021 to 0.081	0.035	0.25	0.059	0.0041 to 0.11	0.073	**0.034**
Fasting serum HDL-cholesterol (mmol/l)	4652	0.023	-0.028 to 0.074	0.018	0.37	-0.032	-0.087 to 0.023	-0.012	0.26
Fasting serum LDL-cholesterol (mmol/l)	3957	0.025	-0.03 to 0.08	0.024	0.38	0.066	0.0072 to 0.12	0.077	**0.028**
Fasting serum triacylglycerol (mmol/l)	4124	-0.014	-0.069 to 0.041	-0.007	0.62	0.050	-0.011 to 0.11	0.043	0.10

The analyses were performed using an additive genetic model. β_{SD} is the effect size estimated from quantile transformed values of the trait and β is the effect size estimated from untransformed values. *p*-values were obtained from the quantile transformation based analyses. ISI, insulin sensitivity index; NA, not available. Nominally significant *p*-values are shown in bold.

ESM Table 4. Association results in up to 1,059 Alaska Native Yup'ik applying a recessive genetic model

	***ITGA1* rs870992**				***LARGE1* rs16993330**			
**Trait (measured unit)**	***n***	**β_{SD}**	**SE**	***p***	***N***	**β_{SD}**	**SE**	***p***
Fasting plasma glucose (mmol/l)	1059	0.059	0.064	0.36	1060	0.049	0.091	0.59
Fasting serum insulin (pmol/l)	855	0.23	0.12	0.058	856	-0.0040	0.20	0.98
HbA_{1C} (%)	960	-0.11	0.048	0.024	963	0.053	0.075	0.48
HOMA-IR	855	0.31	0.17	0.068	856	0.068	0.28	0.81
Weight (kg)	1056	0.036	0.13	0.78	1057	0.15	0.180	0.40
BMI (kg/m^2)	1052	0.056	0.10	0.59	1053	0.17	0.15	0.25
Fasting serum cholesterol (mmol/l)	1059	0.039	0.093	0.67	1060	0.0090	0.13	0.94
Fasting serum HDL-cholesterol (mmol/l)	1054	-0.17	0.093	0.063	1055	0.15	0.13	0.24
Fasting serum LDL-cholesterol (mmol/l)	1054	0.11	0.11	0.35	1055	-0.065	0.16	0.68
Fasting serum triacylglycerol (mmol/l)	1058	0.17	0.15	0.27	1059	0.16	0.21	0.46

The analyses were performed using a recessive genetic model. β_{SD} is the effect size estimated from quantile transformed values of the trait and *p*-values were obtained from these quantile transformation based analyses.

ESM Table 5. Association of *ITGA1* rs870992 and *LARGE1* rs16993330 with type 2 diabetes and metabolic traits in published genome-wide association studies performed using an additive genetic model

	***ITGA1* rs870992**					***LARGE1* rs16993330**							
**Trait**	**β**	**SE**	***p***	**Quantile**	***n***	**β**	**SE**	***p***	**Quantile**	***n***	**Unit β**	**Ancestry**	**Ref.**
Type 2 diabetes	1.058	0.024	**0.018**	**0.025**	152599	0.9964	0.028	0.90		152596	OR	European	[1]
Fasting glucose	0.0022	0.0038	0.56		133010	0.00053	0.0045	0.91		133010	mmol/l	European	[2]
Fasting insulin	-0.00033	0.0044	0.94		108557	0.0029	0.0051	0.58		108557	log(pmol/l)	European	[2]
HbA_{1C}	0.0022	0.0059	0.71		46368	0.0013	0.0075	0.86		46368	%	European	[3]
2h glucose	0.025	0.021	0.22		42854	-0.032	0.023	0.16		42854	mmol/l	European	[2]
BMI	0	0.0054	1		338686	0.0053	0.0064	0.41		321970	IVNT	Mixed	[4]
Height	-0.0038	0.0053	0.47		253151	-0.0023	0.0065	0.73		235006	Z-score	European	[5]
Waist circumference	-6.0×10^{-4}	0.0059	0.91		244161	0.0073	0.0071	0.3		228011	IVNT	Mixed	[6]
Hip circumference	0.0041	0.0063	0.52		227165	0.001	0.0075	0.90		210859	IVNT	Mixed	[6]
WHR	-0.0015	0.0058	0.79		226340	0.0083	0.007	0.23		210370	IVNT	Mixed	[6]
Body-fat percentage	0.005	0.0084	0.55		96950	-0.0079	0.0097	0.41		95590	IVNT	Mixed	[7]
Total cholesterol	0.0278	0.0067	$\mathbf{5.2\times10^{-5}}$	**0.0037**	169088	0.0198	0.0079	**0.026**	**0.035**	180105	IVNT	European	[8]
Triacylglycerol	0.0057	0.0063	0.46		161058	0.0179	0.0074	**0.016**	**0.022**	170650	IVNT	European	[8]
LDL-cholesterol	0.0308	0.0069	$\mathbf{8.5\times10^{-6}}$	**0.0021**	157197	0.0119	0.0082	0.29		166116	IVNT	European	[8]
HDL-cholesterol	0.0033	0.0064	0.57		169801	-0.0046	0.0076	0.54		179989	IVNT	European	[8]

Effects are for *ITGA1* rs870992 G-allele and for *LARGE1* rs16993330 A-allele. Nominally significant *p*-values are shown in bold. Additive genetic model GWASs were queried through online available summary statistics. Quantile is the fraction of *p*-values in the specific set of results, which has lower *p*-value than the queried variant. Type 2 diabetes results were from a recent study by the DIAGRAM Consortium [1] (http://diagram-consortium.org/). Glycemic traits were from MAGIC GWAS [2] (https://www.magicinvestigators.org/). Anthropometric traits were from GIANT Consortium [4-6] (http://portals.broadinstitute.org/collaboration/giant/index.php/GIANT_consortium), while lipid results were from GLGC [8] (http://lipidgenetics.org/). IVNT, inverse-normalized transformation.

References:

[1] Scott RA, Scott LJ, Magi R, et al. (2017) An Expanded Genome-Wide Association Study of Type 2 Diabetes in Europeans. Diabetes 66: 2888-2902

[2] Scott RA, Lagou V, Welch RP, et al. (2012) Large-scale association analyses identify new loci influencing glycemic traits and provide insight into the underlying biological pathways. Nat Genet 44: 991-1005
[3] Soranzo N, Sanna S, Wheeler E, et al. (2010) Common variants at 10 genomic loci influence hemoglobin A(1)(C) levels via glycemic and nonglycemic pathways. Diabetes 59: 3229-3239
[4] Locke AE, Kahali B, Berndt SI, et al. (2015) Genetic studies of body mass index yield new insights for obesity biology. Nature 518: 197-206
[5] Wood AR, Esko T, Yang J, et al. (2014) Defining the role of common variation in the genomic and biological architecture of adult human height. Nat Genet
[6] Shungin D, Winkler TW, Croteau-Chonka DC, et al. (2015) New genetic loci link adipose and insulin biology to body fat distribution. Nature 518: 187-196
[7] Lu Y, Day FR, Gustafsson S, et al. (2016) New loci for body fat percentage reveal link between adiposity and cardiometabolic disease risk. Nat Commun 7: 10495
[8] Willer CJ, Schmidt EM, Sengupta S, et al. (2013) Discovery and refinement of loci associated with lipid levels. Nat Genet 45: 1274-1283

SUPPLEMENTARY FIGURES

a)

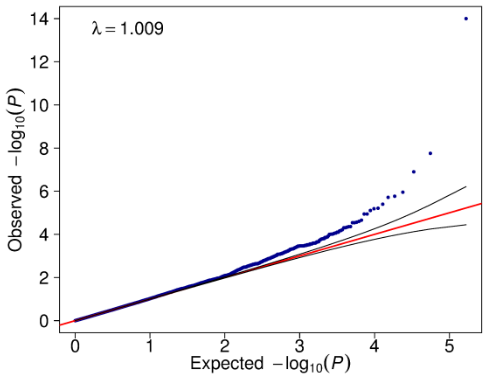

b)

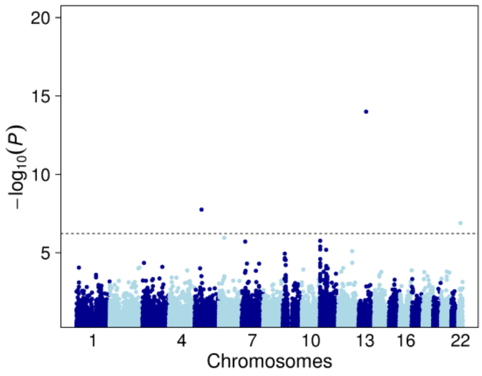

ESM Fig. 1. QQ-plot (a) and Manhattan plot (b) for recessive association analyses of variants on the MetaboChip with type 2 diabetes

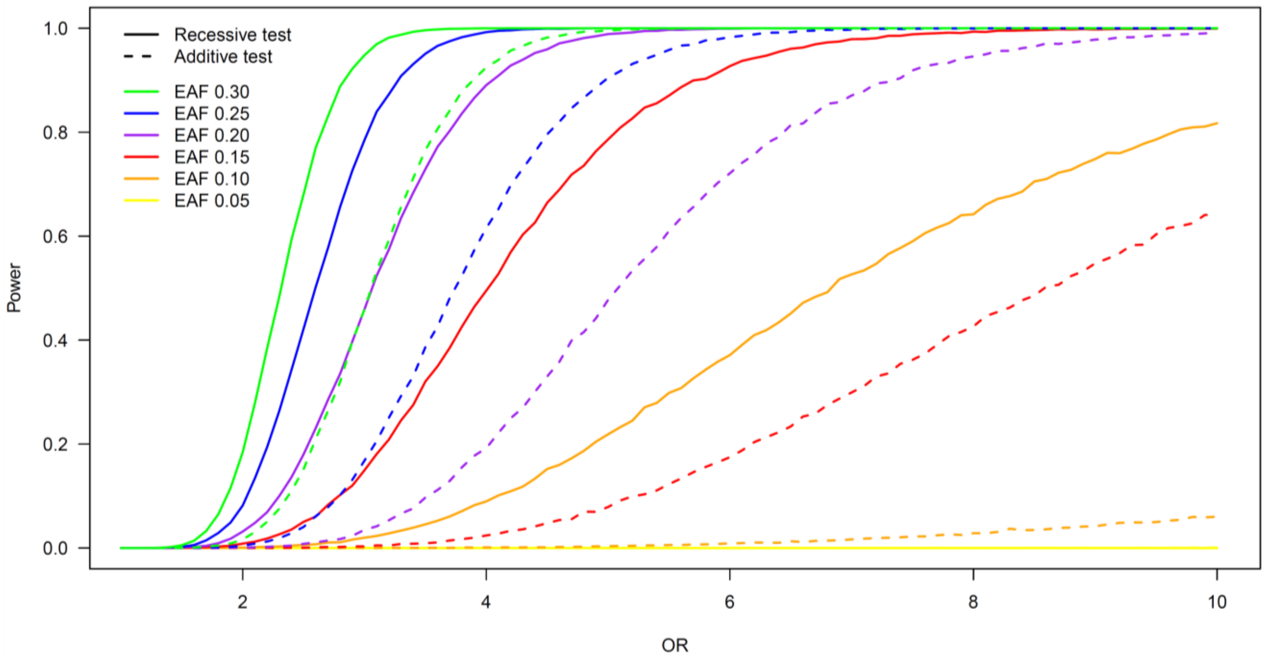

ESM Fig. 2. Statistical power simulation results for a recessive variant applying an additive model based association test (dotted lines) or a recessive model based association test (solid lines). Statistical power is shown as a function of effect measured as OR for each of six different effect allele frequencies (EAF). The power shown is based on simulations where it was assumed that there are 2,948 participants in the study and that the risk of getting type 2 diabetes among participants not carrying two copies of the causal variant is 0.1. As can be seen the recessive model based test has higher power across all EAFs considered here.
